# Editorial: Sex differences in the neurobiology of drug relapse vulnerability

**DOI:** 10.3389/fnbeh.2023.1289459

**Published:** 2023-09-25

**Authors:** Daniel F. Manvich, Jessica A. Loweth, Wendy J. Lynch, Jayme R. McReynolds

**Affiliations:** ^1^Department of Cell Biology and Neuroscience, Rowan University School of Osteopathic Medicine, Stratford, NJ, United States; ^2^Department of Psychiatry and Neurobehavioral Sciences, University of Virginia, Charlottesville, VA, United States; ^3^Department of Pharmacology and Systems Physiology and Center for Addiction Research, University of Cincinnati College of Medicine, Cincinnati, OH, United States

**Keywords:** Substance Use Disorder (SUD), relapse, sex differences, self-administration, neuroimaging, sex hormones

Substance Use Disorders (SUDs) are characterized by high rates of relapse that substantially impede successful treatment outcomes during abstinence (Brandon et al., 2007; Sinha, 2011). Researchers have therefore strived to delineate the factors that drive relapse vulnerability, including a possible role for biological sex. Generally speaking, women transition to SUD faster than men (Towers et al., 2023), report greater drug craving during abstinence (Boykoff et al., 2010; Back et al., 2011; Weinberger et al., 2015; Nicolas et al., 2022), and exhibit an overall higher risk for relapse (Hudson and Stamp, 2011; Becker and Koob, 2016). On the other hand, men seek treatment for SUDs more often than women (SAMHSA, 2022) and account for a greater proportion of fatal drug overdoses (Butelman et al., 2023). These findings raise the possibility that the neurobiological underpinnings of relapse and its outcomes may qualitatively differ between males and females, or alternatively, that there are shared neurobiological mechanisms upon which sex and/or sex-dependent hormones exert a modulatory influence. A better understanding of how relapse neurobiology contrasts between males and females may lead to improved treatments for SUDs, perhaps culminating in sex-specific therapeutic strategies. However, findings from both clinical and preclinical studies examining sex differences have been mixed (Nicolas et al., 2022), with procedural differences often acknowledged as a potential source of incongruence that may underlie disparate results among reports. Moreover, the contributions of sex steroid hormones to observed sex differences have only recently begun to be explored, and investigations into the molecular, cellular, and circuit-level mechanisms that underlie sex differences in drug taking and drug seeking behaviors are lacking.

In this focused Research Topic, we are pleased to present five original research articles – three preclinical studies, and two clinical studies – that help to address these gaps in knowledge. The Research Topic articles describe impacts of biological sex on the direct reinforcing effects of drugs with abuse liability as well as sex-dependent differences in drug craving and/or seeking assessed at various time points following cessation of drug use. The studies span several drug classes (psychostimulants, opioids, alcohol, and nicotine) and a subset also explore the contributions of fluctuating sex hormones in females.

In the first preclinical study, Scott et al. investigated whether sex and/or estrous cyclicity modulates the effects of the 5-HT_1B_ receptor agonist CP94253 on IV cocaine self-administration in rats. Pretreatment with CP94253 enhanced cocaine intake and break points in both males and females under a progressive-ratio schedule of reinforcement and reduced responding for high cocaine doses under a fixed-ratio schedule of reinforcement in females (a finding previously demonstrated in males), effects that are both consistent with potentiated cocaine reinforcing efficacy. Interestingly, the modulation of cocaine self-administration under the FR schedule was estrous cycle-dependent, as CP94253 was ineffective in female rats during metestrus.

Blair Towers et al. explored whether incubated cocaine-craving develops at different rates in male vs. female rats, and whether changes in gene expression previously linked to incubated cocaine-craving in male rats occur in females. Following 10 days of extended-access cocaine self-administration, cue-induced cocaine-seeking behavior significantly intensified across withdrawal time points in males, but was prematurely elevated in females as early as withdrawal day 2 and did not increase further. There were also dramatic sex differences in molecular adaptations within the dorsomedial prefrontal cortex across withdrawal. *Grin1* expression increased in both sexes but at different withdrawal time points, whereas expression of other markers (*Grin2a, Grin2b, Bdnf-IV*) were altered exclusively in males, suggesting that neurobiological underpinnings of incubated cocaine-craving may differ between sexes.

Hinds et al. examined whether sex and/or estrous cyclicity alters the primary reinforcing effects of IV oxycodone or the expression of oxycodone-seeking behavior. Male and female rats did not differ in their average rate of IV oxycodone self-administration or intake under a simple fixed-ratio schedule, however oxycodone-maintained responding was significantly lower in females during proestrus/estrus phases of the estrous cycle as compared to metestrus/diestrus. By contrast, there were neither sex-dependent nor cycle-dependent effects on levels of cue-induced oxycodone seeking.

Transitioning to clinical studies, Zakiniaeiz et al. compared the interactive relationships between smoking history, plasma sex steroid hormone levels, and D_2/3_ receptor (D_2/3_R) availability in the dorsolateral prefrontal cortex between men and women. Consistent with prior reports, estradiol levels were significantly increased in male smokers but trended toward lower levels in female smokers as compared to same-sex healthy controls. Intriguingly, sex steroid hormone levels were associated with altered D_2/3_R availability in a sex-dependent manner, as low D_2/3_R availability in women was related to significantly lower estradiol levels and a trend for increased free testosterone, whereas hormonal levels were not associated with D_2/3_R availability in men. The authors suggest that women may experience greater difficulty with smoking cessation due to sex-specific interactive effects of hormones and nicotine on the dopamine system and cognitive function.

Finally, Gerhardt et al. used functional magnetic resonance imaging to measure neural reactivity to alcohol-associated imagery in a cohort of men and women with diagnosed Alcohol Use Disorder (AUD). To their surprise, and in contrast to findings in younger social drinkers, the authors did not detect significant sex differences in alcohol cue reactivity in any brain regions of interest, although reactivity trended toward higher levels in females. They suggest that alcohol cue reactivity may change over time such that sex differences which are apparent during early stages of alcohol use may fade or altogether disappear with more severe alcohol use.

These five manuscripts add to a growing literature that is furthering our understanding of the neurobiology of drug relapse and how it is impacted by biological sex, and also admirably describe important future directions that will help facilitate new discoveries. For example, longitudinal studies in human SUD patients could ascertain whether the impact of sex on drug reinforcement and relapse vulnerability changes during transitions from initial to prolonged drug use, early to late abstinence, etc. Several authors highlight the need to better characterize the mechanisms by which ovarian hormone fluctuations alter SUD outcomes and discuss important methodological considerations that may facilitate the detection of sex differences and reproductive cycle influences on drug taking and drug-seeking behaviors in preclinical experiments. Finally, the cellular and molecular mechanisms through which sex and sex-dependent hormonal states impact relapse susceptibility have only recently been explored, with some examples provided in this Research Topic. Future research should build upon these advances and recommendations, using multidisciplinary approaches and cutting-edge technologies, so that new treatments with improved efficacy can be developed for SUDs.

## Author contributions

DM: Writing—original draft, Writing—review and editing. JL: Writing—review and editing. WL: Writing—review and editing. JM: Writing—review and editing.
